# Immunity of turbot Induced by inactivated vaccine of *Aeromonas salmonicida* from the perspective of DNA methylation

**DOI:** 10.3389/fimmu.2023.1124322

**Published:** 2023-02-07

**Authors:** Yingrui Li, Lin Su, Xiaofei Liu, Huimin Guo, Shun Zhou, Yunji Xiu

**Affiliations:** Institute of Marine Science and Engineering, Qingdao Agricultural University, Qingdao, China

**Keywords:** DNA methylation, *Scophthalmus maximus*, *Aeromonas salmonicida*, inactivated vaccine, Whole Genome Bisulfite Sequencing, transcriptome sequencing

## Abstract

**Introduction:**

DNA methylation was one of the most important modification in epigenetics and played an important role in immune response. Since the introduction of *Scophthalmus maximus*, the scale of breeding has continued to expand, during which diseases caused by various bacteria, viruses and parasites have become increasingly serious. Therefore, the inactivated vaccines have been widely researched and used in the field of aquatic products with its unique advantages. However, the immune mechanism that occurred in turbot after immunization with inactivated vaccine of *Aeromonas salmonicida* was not clear.

**Methods:**

In this study, differentially methylated regions (DMRs) were screened by Whole Genome Bisulfite Sequencing (WGBS) and significantly differentially expressed genes (DEGs) were screened by Transcriptome sequencing. Double luciferase report assay and DNA pull-down assay were further verified the DNA methylation state of the gene promoter region affected genes transcriptional activity after immunization with inactivated vaccine of *Aeromonas salmonicida*.

**Results:**

A total of 8149 differentially methylated regions (DMRs) were screened, in which there were many immune-related genes with altered DNA methylation status. Meanwhile, 386 significantly differentially expressed genes (DEGs) were identified, many of which were significantly enriched in Toll-like receptor signaling pathway, NOD-like receptor signaling pathway and C-type lectin receptor signaling pathway. Combined analysis of WGBS results and RNA-seq results, a total of 9 DMRs of negatively regulated genes are located in the promoter region, including 2 hypermethylated genes with lower expression and 7 hypomethylated genes with higher expression. Then, two immune-related genes C5a anaphylatoxin chemotactic receptor 1-like (*C5ar1-Like*) and Eosinophil peroxidase-like *(EPX-Like)*, were screened to explore the regulation mechanism of DNA methylation modification on their expression level. Moreover, the DNA methylation state of the gene promoter region affected genes transcriptional activity by inhibiting the binding of transcription factors, which lead to changes in the expression level of the gene.

**Discussion:**

We jointly analyzed WGBS and RNA-seq results and revealed the immune mechanism that occurred in turbot after immunized with inactivated vaccine of *A. salmonicida* from the perspective of DNA methylation.

## 1 Introduction

Epigenetics, including DNA methylation, histone modification, and the regulation of noncoding RNAs, could cause heritable change in gene function without changes in DNA sequence, which produce heritable phenotypic changes eventually. As the most widely characterized epigenetic modification, DNA methylation was produced *via* 5-methylcytosine (5mC) binding a methyl group (CH_3_) to the cytosine with the help of DNA methyltransferases (DNMTs) (1). DNA methylation has been widely reported to regulate gene expression, which was mostly conducted by inhibiting the binding of transcription factors on the promoters. Previous studies confirmed that DNA methylation was involved in immune response modulation. Abnormal DNA methylation was closely related to the occurrence and development of many cancers and immune diseases (2). With the development of research, the role of DNA methylation modification on immune process of aquatic animals has also been extensively explored. For example, *Ctenopharyngodon idella Retinoic acid-inducible gene I* (*CiRIG-I*) that was modified by methylation was extremely related with the resistance to grass carp reovirus (GCRV) and may be a negative regulator of *CiRIG-I* antiviral transcription (3). Besides, analysis of genome-wide DNA methylation in the spleen of resistant and susceptible Nile tilapia (*Oreochromis niloticus*) infected with *Streptococcus agalactiae* found that the methylation status was higher in the spleen samples from resistant fish than in the susceptible group (4). The above-mentioned studies indicate that DNA methylation participated in the immune defense mechanism of aquatic animals through regulating gene expression.

*Scophthalmus maximus*, an economically valuable flatfish, is widely distributed from Norway to the Mediterranean and China (5). The turbot is characterized with the advantages of low temperature resistance, grows fast and tastes delicious, which makes the turbot aquaculture industry develop rapidly (6). However, with the expansion of culture scale, the diseases caused by various bacteria, viruses, parasites and other pathogens have become more and more serious, causing great threats and destruction to the breeding industry. Compared with parasitic diseases and viral diseases, bacterial diseases have caused serious economic losses due to their wide spread, fast spread, strong pathogenicity and high mortality. For example, turbot could be infected by *A. salmonicida* to cause furunculosis (7), resulting in ulcerate in the infected skin area, oral bleeding, ulceration of fin edges and subcutaneous hemorrhage at the bottom, which eventually lead to the death of turbot. The traditional prevention and control methods of bacterial diseases in the turbot breeding process mainly relied on antibiotics and chemical drugs. However, the frequent use of these drugs could lead to the production of drug-resistant bacteria and immunosuppression of fish. In addition, the residual drugs existing in the turbot and in the aquaculture water might threaten human health. In fact, in the process of aquaculture, inactivated vaccines have been widely studied for its advantages such as high security, easy preparation and storage, non-pollution and easy production of combined vaccines or multivalent vaccines. On the one hand, vaccination significantly reduced the relative abundance values of potential opportunistic pathogens such as Aeromonas, Escherichia-Shigella, and Acinetobacter in teleost. On the other hand, combined with the enhancement of immune function after vaccination, inactivated Aeromonas vaccination had a protective effect against the pathogen infection of teleost. In the previous research, the relative immune protection rate of inactivated vaccine of *A. salmonicide* was up to 72.72% after inoculation with the *A. salmonicide* inactivated vaccine. Besides, turbot could achieve effective immune protection and induced immune responses by the immunization of inactivated vaccine of *A. salmonicida* (8).

The regulation of DNA methylation on immune response and the immune mechanism triggered by inactivated vaccines were widely reported in aquatic animals. For example, the immune-related genes of aquatic animals could be effectively activated by inactivated vaccines. Bacteria-mediated immune response was often closely related to aberrant DNA methylation. According to the current research, the potential link between vaccines and DNA methylation has emerged. Previous studies have found that Bacillus Calmette Guerin Vaccine injection induced a persistent change in DNA methylation, which was involved in antibacterial response (9) and pneumococcal vaccines leaded to changes in DNA methylation, which was associated with immune response activation (10). Balb/c mice vaccinated with a protective vaccine could cause changes in the DNA methylation level of gene promoters in livers, which ultimately improve the survival of the mice after infection with *P. chabaudi* (11). However, the effect of inactivated vaccines on DNA methylation patterns in fish and the mechanism by which DNA methylation was involved in the immune response *in vivo* were unclear.

In this study, whole-genome bisulfite sequencing (WGBS) and transcriptome sequencing were performed on turbot kidney tissue during the immunization of *A. salmonicide* inactivated vaccine, after which the expression changes of immune-related genes and genome-wide DNA methylation profiles were revealed. The sequencing results of the two groups were jointly analyzed and obtained differentially methylated regions (DMRs) and differentially expressed genes (DEGs) related to immune response. Besides, qRT-PCR and the double luciferase reporter assay was conducted and confirmed that DNA methylation inhibited gene expression by repressing the transcriptional activity of gene promoters. DNA pull down and Mass Spectrometry (MS) illustrated that DNA methylation effected binding of transcription factors to immune-related gene the and further influenced the expression of immune-related gene. In a word, our study revealed the potential relationship between DNA methylation levels and gene expression levels during the immunization of inactivated vaccines, which reveals the regulatory effect of inactivated vaccine on the immune mechanism of turbot from the perspective of DNA methylation, and provides new ideas for disease resistance breeding of turbot.

## 2 Methods and materials

### 2.1 Experimental fish, vaccine immunity and sample collection

Healthy turbot with an average weight of 80 ± 10 g was ordered from Haiyang Yellow Sea Aquatic Product Co.,Ltd., and kept in the laboratory until the inactivated vaccine of *A. salmonicida* was injected. Preparation of inactivated vaccines was performed as described previously (8). The formaldehyde-inactivated vaccine of *A. salmonicida* (1×10^9^ CFU/ml) was prepared at a concentration of 5‰ at 4 °C for 24 h. The turbot was immunized by intraperitoneal injected with 0.1 ml of inactivated vaccine (LD_50 =_ 2.63×10^6^ CFU/mL). Nine healthy turbot kidney tissue were randomly selected as the control group (named as NVSm) before vaccination, and nine challenged kidney tissue were randomly selected as the experimental group (named as AsVSm) after 30 days of inactivated vaccine immunization. In order to ensure the authenticity of the experimental results, we mixed the kidney tissue of 3 fish as a sample, and each group had 3 biological replicates. The tissue samples were quickly frozen in liquid nitrogen and then transferred to -80 °C refrigerator for storage.

### 2.2 Transcriptome sequencing and bioinformatics analysis

The TRIzol reagents were used to extract the total RNA from turbot kidney tissue in accordance with the manufacturer’s instructions (Invitrogen, USA). After purification and quantification, a total of 1 µg of RNA per sample was utilized for the RNA sample preparations. Using the NEBNext^®^ UltraTM RNA Library Prep Kit for Illumina^®^ (NEB, USA) in accordance with the manufacturer’s instructions, sequencing libraries were generated. The mRNA containing polyA tails was enhanced using oligo (dT) magnetic beads, and the resulting mRNA was then randomly interrupted in NEB Fragmentation Buffer. The M-MuLV reverse transcriptase system was used to create the first strand of cDNA using fragmented mRNA as a template and random oligonucleotides as primers. The RNA strand was then broken down using RNaseH, and the DNA polymerase I apparatus was utilized to create the second strand of cDNA utilizing dNTPs as the starting material. The end of the purified double-stranded cDNA was repaired, an A-tail was inserted, and a sequencing adaptor was attached. To create the library, the 250–300 bp cDNA was first screened with AMPure XP beads, followed by PCR amplification and further purification of the PCR products with AMPure XP beads. Use a Qubit2.0 Fluorometer to first measure the library, then diluted it to 1.5 ng/µL. The Agilent 2100 bioanalyzer then identified the insert size of the library and carried out qRT-PCR to precisely measure the library’s effective concentration and guarantee the library’s quality. After the library was validated, the various libraries were combined based on the intended offline data volume and effective concentration. Following that, Illumina sequencing was carried out, and a 150bp paired-end reading was produced.

Raw data were initially treated using in-house perl scripts to gain clean reads. The clean data for Q20, Q30, and GC content were evaluated simultaneously. The clear data with high-quality served as the foundation for all downstream studies. Hisat2 v2.0.5 was used to build index of the reference genome and aligned paired-end clean reads to the reference genome. The number of reads that were mapped to each gene was counted using FeatureCounts v1.5.0-p3. The length of each gene and the number of reads mapped to this gene were used to calculate the FPKM of each gene. Using the DESeq2 R software (1.16.1), differential expression analysis of two groups (three biological replicates per condition) was carried out. The Benjamini and Hochberg method for reducing the false discovery rate was used to modify the resulting *P*-values. Genes identified by DESeq2 as having differential expression were those with an adjusted *P*-value < 0.05. The clusterProfiler R package was used to implement the Gene Ontology (GO) enrichment analysis of DEGs, and gene length bias was addressed. GO terms were deemed to be substantially enriched by differentially expressed genes when the adjusted *P*-value < 0.05. Additionally, the statistical enrichment of differentially expressed genes in KEGG pathways was examined using the clusterProfiler R program. WGBS Library Construction, Sequencing and Bioinformatics Analysis

### 2.3 WGBS library construction, sequencing and bioinformatics analysis

Using the OMEGA Tissue DNA Kit, genomic DNA was extracted from the harvested kidney tissue and examined on agarose gels for deterioration and contamination. Using the Qubit^®^ 2.0 Flurometer (Life Technologies, CA, USA) and NanoPhotometer^®^ spectrophotometer (IMPLEN, CA, USA), respectively, the DNA purity and concentration were assessed. The subsequent procedure form a DNA library from the genomic DNA of the turbot kidney tissue. 5.2 μg of genomic DNA spiked with 26 ng of lambda DNA was sonicated into 200-300 bp-sized pieces using a Covaris S220. Then, using the EZ DNA Methylation-GoldTM Kit (Zymo Research, China), these DNA fragments were subjected to two rounds of bisulfite treatment. The resultant single-strand DNA fragments were then amplified using KAPA HiFi HotStart Uracil + ReadyMix (2X). The Agilent Bioanalyzer 2100 system was used to measure the insert size, and the DNA library concentration was measured using the Qubit^®^ 2.0 Flurometer (Life Technologies, CA, USA) and quantitative PCR.

On the Illumina Hiseq 4000 platform, the library preparations were sequenced, and 150 bp paired-end reads were produced. The basis of information analysis is the quality control of the data to produce qualified data. Using FastQC (fastqc v0.11.5), basic statistics on the caliber of the raw readings were calculated. All following studies were dependent on the quality of the cleandata reads, which were first obtained by pre-processing the raw reads sequence using the parameters program. Basic statistics on the quality of the clean data readings were performed using FastQC. The reads to a reference genome were then carried out using Bismark software (version 0.16.3) (12). The two alignment procedures created a unique best alignment of sequence reads, and by comparing it to the typical genomic sequence, it is possible to deduce the methylation status of any place containing cytosine in the read. For IGV browser display, the methylation extractor data were converted to bigWig format (13). The sequence was then separated into several bins that were each 10 kb in size to determine the sequence’s methylation level (14).

The DMRs were located using the DSS program (15). We define genes associated to DMRs as genes whose genomic area (from TSS to TES) or the promoter region (upstream 2kb from the TSS) coincides with DMRs in accordance with the distribution of DMRs in the genome. The GOseq R package (16), which corrects for gene length bias, was then used to execute Gene Ontology (GO) enrichment analysis of genes associated with DMRs. DMR-related genes were thought to substantially enrich GO terms with a corrected *P*-value < 0.05. The statistical enrichment of DMR-related genes in KEGG pathways was examined using KOBAS software (17).

### 2.4 Quantitative real-time PCR

Using the BIORAD CFX96 Touch fluorescence quantitative PCR equipment, qPCR was carried out using *β*-actin as an internal standard after the mRNA was transformed to cDNA using a PrimeScriptTM RT reagent Kit (TaKaRa, China) (Bio-Rad Laboratories, CA, USA). The reaction system was composed of 10 L of ddH2O, 5 L of TB Green Premix Ex Taq II (2×)(Takara, China), 0.4 L of a particular forward/reverse primer, and 0.8 L of cDNA. The primers were listed in **Table 1**. The following were the conditions for quantitative PCR: 95 °C for 30s, 35 cycle of 95 °C for 5 s and 60 °C for 1 min. The following conditions were used during the melting curve to confirm the amplicons’ specificity: 95 °C for 10 s, 65 °C for 5 s and up to 95 °C at a rate of 0.5 °C/cycle. Each sample was made three biological replicates to reduce deviation. Using the 2^-△△Ct^ method, gene expression was evaluated in relation to β-actin expression.

**Table 1:** List of the primers used in this manuscript.

Gene name	Primer name	Primer sequence (5’ – 3’)	Assay
*C5ar1-Like*	*C5ar1-Like*-qRT-F	TCGTGGGATTCTTCCTCCCT	qPCR
	*C5ar1-Like*-qRT-R	GGAAGTCCAAGACGTGCAGA	
*EPX-Like*	*EPX-Like*-qRT-F	ACCAGAACCACTACAGCACG	
	*EPX-Like*-qRT-R	TTCAGCCGGAGAAGTGTGTC	
Sm*β*-actin	Sm*β*-actin-qRT-F	AATGAGCTGAGAGTTGCCCC	
	Sm*β*-actin-qRT-R	AGCTTGGATGGCAACGTACA	
*C5ar1-Like*	*C5ar1-Like*-BSP-F	ATTTTAATTTATAGGTTTAGTGGT	Bisulfite sequencing PCR
	*C5ar1-Like*-BSP-R	CAAATAAATATTATAAACAAATTATAAAC	
*EPX-Like*	*EPX-Like*-BSP-F	GTGTTTTTGTAAATTTTTTTAAAAA	
	*EPX-Like*-BSP-R	AAATTTCCTTTAACACAAAAAAAA	
C5ar1-Like	C5ar1-Like-F1	CGGGGTACCGAGTTTATATTTGGGA	Dual luciferase report assay
	C5ar1-Like-R1	CCCAAGCTTCTTACAGGCTCAGTGG	
	C5ar1-Like-F2	CGGGGTACCGTTGTAAGCAGGTTGTAG	
	C5ar1-Like-R2	CCCAAGCTTTATTTCAACTTACAGGCT	
EPX-Like	EPX-Like-F1	CGGGGTACCGCCATAAGCACAAGAAAACTCCC	
	EPX-Like-R1	CCCAAGCTTTGTCCCTGTAAACCCCCCCAAAA	
	EPX-Like-F2	CGGGGTACCTCAAAATGTAAGAAACTGCT	
	EPX-Like-R2	CCCAAGCTTGAAAATCTCATTACCAGTGT	
	EPX-Like-F3	CGGGGTACCCTTCACCTGGACAACCCT	
	EPX-Like-R3	CCCAAGCTTAAATCTCATTACCAGTGT	
pGL3-basic	RVP3	CTAGCAAAATAGGCTGTCCC	
	GLP2	CTTTATGTTTTTGGCGTCTTCCA	
SmC5ar1-Like-F	CGTGTGCGTCCGGGACGGGCGAAGTATAAAAACTCACCGAAAAGCAGAAGAGCTCCATAACTTCCAGCGAGCGGTGAGGACACAACGTTGACCG		DNA pull down
SmC5ar1-Like-R	CGGTCAACGTTGTGTCCTCACCGCTCGCTGGAAGTTATGGAGCTCTTCTGCTTTTCGGTGAGTTTTTATACTTCGCCCGTCCCGGACGCACACG		
met-SmC5ar1-Like methylation probes	CGTGTGCGTCCGGGACGGGCGAAGTATAAAAACTCACCGAAAAGCAGAAGAGCTCCATAACTTCCAGCGAGCGGTGAGGACACAACGTTGACCG		

### 2.5 Bisulfite sequencing PCR

The Tissue DNA Kit (OMEGA, China) was used to extract genomic DNA from kidney samples from the AsVSm group and NVSm group, each group contains 3 biological repetition. The genomic DNA of the three samples of the AsVSm group and the NVSm group were mixed in equal amounts (1 μg) and used as a template. According to the manufacturer’s instructions, the EZ DNA Methylation-Gold™ Kit (ZYMO RESEARCH, USA) was used for bisulfite modification. The reaction system was 50 μL including methylation modified DNA 4 μL, 10×EpiTaq PCR Buffer (Mg^2+^ free) 5 μL, 25mM MgCl_2_ 6 μL, dNTP Mixture 4 μL, BSP forward/reverse primer 5 μL, TaKaRa EpiTaq HS (5 U/μL) (TaKaRa, China) 0.3 μL and ddH_2_O. The BSP primers were produced using the MethPrimer program, which is available online at http://www.urogene.org/methprimer/. **Table 1** contains the sequence details. The following steps were taken in order to execute the BS-PCR: 3 min at 98°C, then 35 cycles of 10 s of denaturation at 98°C, 30 s of annealing at 55°C, and 30 s of extension at 72°C, with a final extension at 72°C for 7 min. The PCR products were assembled on the pEASY-T1 Cloning Vector after being gel purified using the TaKaRa MiniBEST Agarose Gel DNA Extraction Kit Ver.4.0 (TaKaRa, China) (TransGen, China). The recombinant plasmid was transferred into *Trans1*-T1 competent cells (TransGen, China) according to the manufacturer’s instructions of the *pEASY*-T1 Cloning Kit (TransGen, China). At least 10 different positive clones of each subject were randomly selected for sequencing. The final sequence results were processed by online BiQ-Analyzer (https://biq-analyzer.bioinf.mpi-inf.mpg.de/ ).

### 2.6 *In vitro* methylation modification and dual luciferase reporter assay

The dual luciferase reporter assay was used to verify the effect of DNA methylation modification on the gene expression. The DMR from the promoter region was amplified with different length to explore the effect of different promoter fragments on gene expression. Primer 5 was used to design specific primers (**Table 1**). The 12.5 μL 2×Taq PCR Mix, 2μL of DNA Template, 0.5μL of a particular forward/reverse primer, and 25μL of ddH_2_O made up the PCR reaction system. The following steps were used to carry out the PCR: 5 min at 94°C, then 34 cycles of 30 s of denaturation at 94°C, 30 s of annealing at 55°C, and 1 min of extension at 72°C, with a final extension lasting 7 min at 72°C. The qualified target fragments were then retrieved after the 1% agarose gel electrophoresis was used to confirm the PCR results. The construction of dual luciferase reporter recombinant plasmid was constructed as follows: First of all, the restriction enzymes *Hind* III and *Kpn* I were used to double digest the target fragment and the empty pGL3-basic vector. Then, the recovered and purified vector and the target fragment were ligated at a molar ratio of 1:10 using T4 DNA ligase overnight at 16 °C to obtain recombinant plasmids. The recombinant plasmids were transferred into *Trans1*-T1 competent cells according to the previous method, and then the positive clones were selected and sequenced. The competent cells were cultivated overnight to amplify the recombinant plasmid verified by sequencing, and then extract the endotoxin-free recombinant plasmid according to the manufacturer’s instructions of Endo-free Plasmid Mini Kit II(OMEGA, China).

CpG methyltransferase (M.Sss I) was used to modify recombinant plasmids methylation *in vitro*. M.Sss I can specifically mimic the modification mode of higher eukaryotic genomes, which will specifically modify the “CG” site on double-stranded DNA with the help of S-adenosylmethionine (SAM). The reaction system was as follows: 1 μg recombinant plasmid, 1 μL M.Sss I methylase, 2 μL SAM (1600 μM), 2 μL 10×NEBuffer 2 and added with Nuclease-free water for a final volume of 20 μL. Then, the reaction system was incubated at 37 °C for 1 h and terminated at 65 °C for 20 min. The *Hpa* II restriction endonuclease was used to verify the *in vitro* methylation modification of recombinant plasmid. The *Hpa* II can cut the unmethylated CG site, and the *in vitro* methylation modification of recombinant plasmid can be identified according to whether the digested product produces fragments of different lengths. The reaction system was as follows: 1 μL *Hpa* II, 1 μg recombinant plasmids with methylation modification, 5 μL 10×NEBuffer and added with ddH_2_O for a final volume of 50 μL. Then, the restriction enzyme reaction system was incubated at 37 °C for 15 min and terminated at 80 °C for 20 min. The results of methylation modification *in vitro* were verified by 1% agarose gel electrophoresis.

*In vitro*, the recombinant plasmids methylation was transfected into HEK293 cells according to the instructions of the manufacturer of the Lipo6000™ Transfection Reagent (Beyotime, China). The pRL-TK plasmid was used as an internal reference plasmid. The recombinant plasmids methylation and pRL-TK plasmid were co-transfected into HEK293T cells and cultured for 48h to detect the fluorescence activity. At the same time, the unmethylated recombinant plasmid was co-transfected with pRL-TK plasmid as a control, and the pGL3-basic empty vector was co-transfected with pRL-TK plasmid as a negative control, with 3 replicates in each group. According to the instructions provided by the manufacturer of the Dual-LumiTM II Luciferase Reporter Gene Assay Kit, Renilla luciferase and firefly luciferase activity were found in the cell lysate (Beyotime, China). The activity of renilla luciferase was used to standardize the activity of firefly luciferase in individual transfections.

### 2.7 DNA pull down and mass spectrometry

The protein potentially binds to the DNA probe of DMR was identified by the DNA pull down technology. Using DMR of *C5ar1-Like* gene as the target sequence, the primers were designed to synthesize specific probes (control group, SmC5ar1-Like) and methylation probes (experimental group, met-SmC5ar1-Like) respectively, and labeled with biotin (**Table 1**). A 500 µL system comprised of 200 pmol biotin-labeled DNA, nucleic acid incubation buffer, and beaver magnetic beads was incubated for 1 hour at room temperature to create a DNA-magnetic bead combination. DNA-magnetic bead complex was magnetically separated after being rinsed twice with pre-cooled nucleic acid incubation buffer and protein incubation buffer. The HEK293 cell protein extract and the protein incubation buffer were formed into a 500 µL system and then incubated with DNA-magnetic bead complex at 4 °C overnight to form a protein-DNA-magnetic bead complex. The protein-DNA-magnetic bead complex was centrifuged and rinsed with pre-cooled protein incubation buffer. The precipitate added100 µL of protein elution buffer. Then, the mixture was heated for 5 min in a 95°C water bath and centrifuged at 12000 rpm for 15min. Supernatant was taken for WB and MS analysis.

The protein solution of met-SmC5ar1-Like experimental group and SmC5ar1-Like control group were subjected to MS respectively to screen the differential proteins, after which the different proteins in the experimental group was selected. The gel was chopped and decolorized with a decolorizing solution (50mM NH4HCO3 and ACN (1:1)), and was dehydrated by adding acetonitrile and vacuum dried. The proteins were then alkylated by 55 mM IAM in the darkroom for 1 hour after being reduced with DTT at 37°C for 1 hour. After dehydration, 0.01 µg/µL Trypsin was added and the mixture was reacted at 37 °C overnight and centrifuged to collect the supernatant of the enzymatic hydrolysis. The remaining micelles were added to acetonitrile and vortexed for 5 min, and centrifuged to collect the supernatant of the enzymatic hydrolysis. In addition, 0.1% FA was added to the remaining micelles and collected the supernatant of the enzyme hydrolysis. All supernatants were collected in the same centrifugal tube for vacuum lyophilization and stored at -20°C.The mobile phase A liquid (100% mass spectrometry water, 0.1% formic acid) and B liquid (80% acetonitrile, 0.1% formic acid) were prepared, respectively. The lyophilized powder were dissolved in 10 µL of A liquid, and centrifuged at 14000 g for 20 min at 4 °C. Then, 1 µg of the supernatant was collected for liquid quality detection. Data acquisition occurred by MS. The MS1 scans were acquired over a range of 300–1500 m/z at a resolution of 120 000 at m/z 200, with an automated gain control (AGC) target of 2×10^5^, and a maximum ion injection time of 50 ms. The precursor ions were fragmented using the high energy collision cracking (HCD) method, and then detected by the secondary mass spectrometry. The MS2 precursors was set to 15000 (200 m/z) with the AGC of 5×10^4^, and the maximum ion injection time of 45 ms. Precursors were fragmented by high-energy collision dissociation at a collision energy of 30% to generate the original data of the MS detection.

## 3 Result

### 3.1 Analysis of transcriptome sequencing results

**Figure 1:**
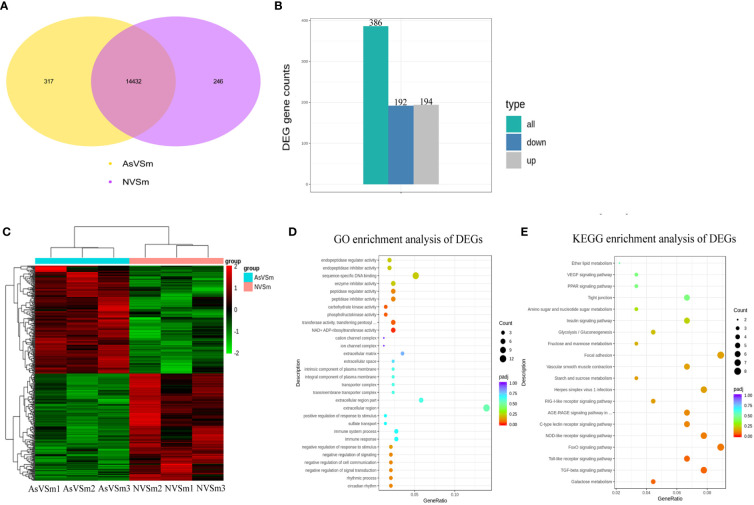
Screening of differential genes by transcriptome sequencing. **(A)** Venn diagram showed differential gene in the comparison of AsVSm and NVSm. **(B)** The bar chart showed statistics of the number of significantly DEGs. **(C)** Clustering heat map of significantly DEGs. The abscissa represents the sample name, and the ordinate represents the value of fpkm after homogenization. Red means high expression and green means low expression. **(D)** GO enrichment analysis of significantly DEGs. **(E)** Scatter plot of KEGG enrichment. The most significantly enriched top 20 pathways of significantly DEGs in AsVSm vs NVSm.

Transcriptome sequencing was performed on the samples of the AsVSm group and the NVSm group to explore the impact of the inactivated vaccine of *A. salmonicida* on the immunity of turbot. The raw reads of the AsVSm group and NVSm group samples were filtered to obtain a total of 32.26G and 28.42G of clean reads, respectively (**Table 2**). Sequencing error rate distribution check results reflected that the quality of sequencing data meets the requirements of subsequent analysis. The clean reads were aligned to the reference genome, and the results were shown in **Table 3**. More than 91.11% of the clean reads were aligned to the reference genome in all 6 samples, and the unique alignment rate was between 88.62% and 89.2%. It shows that the tested species was consistent with the reference genome, the sequencing results were very accurate and credible. The gene expression level was compared to screen the DEGs after quantitative analysis of the gene expression level of the AsVSm group and NVSm group samples. A total of 14,995 differential genes were screened, including 14,432 genes shared by the AsVSm group and the NVSm group, as well as 317 unique genes of the AsVSm group and 246 unique genes of the NVSm group (**Figure 1A**). Then, using |log2 (FoldChange)|> 0 & padj <0.05 as the screening threshold, 386 significantly DEGs were screened, including 194 genes with up-regulation and 192 genes with down-regulation (AsVSm vs NVSm) (**Figure 1B**). The cluster analysis was carried out by taking the union of significantly DEGs of each comparison group, and the results were shown in **Figure 1C**. The gene expression patterns between biologically duplicated samples were very similar, while the expression patterns of DEGs between the AsVSm group and NVSm group showed significant differences.

**Table 2:** The summary of sample data quality.

Sample	Raw_reads	Clean_reads	Clean_bases	Error_rate(%)	Q30(%)	GC_pct(%)
NVSm_1	52550964	51640142	7.75G	0.02	94.99	50.3
NVSm_2	78598000	77295498	11.59G	0.02	95.17	51.08
NVSm_3	61550178	60511312	9.08G	0.02	94.69	49.46
AsVSm_1	69415734	68532074	10.28G	0.02	94.72	50.74
AsVSm_2	72174578	71086780	10.66G	0.02	95.21	50.73
AsVSm_3	76623394	75498176	11.32G	0.02	95.08	50.69

**Table 3:** The statistics of comparison samples and reference genomes.

Sample	NVSm_1	NVSm_2	NVSm_3	AsVSm_1	AsVSm_2	AsVSm_3
total_reads	51640142	77295498	60511312	68532074	71086780	75498176
total_map	47135061 (91.28%)	70962598 (91.81%)	55134031 (91.11%)	62631841 (91.39%)	65200633 (91.72%)	69181075 (91.63%)
unique_map	45814685 (88.72%)	68945071 (89.2%)	53651116 (88.66%)	60736194 (88.62%)	63292839 (89.04%)	67204082 (89.01%)

In order to discover the functions of DEGs and the interaction mechanism between DEGs, the functional enrichment analysis was carried out. The GO enrichment analysis showed that in terms of biological process, DEGs were significantly enriched in negative regulation of signal transduction, negative regulation of response to stimulus, immune response and immune system process. In terms of cellular component, DEGs were significantly enriched in extracellular region, transmembrane transporter complex and integral component of plasma membrane. In terms of molecular function, DEGs were significantly enriched in NAD^+^ ADP-ribosyltransferase activity, transferase activity, transferring pentosyl groups, phosphofructokinase activity and carbohydrate kinase activity (**Figure 1D**). These results showed that the genes involved in immune response and signal transduction were enriched in turbot after immunization with inactivated vaccine of *A. salmonicida*, which promoted the activation of immune-related signaling pathways and the regulation of immune response. The results of KEGG enrichment showed that DEGs were significantly enriched in a number of immune-related signaling pathways, such as the TGF-beta signaling pathway, Toll-like receptor signaling pathway, NOD-like receptor signaling pathway and C-type lectin receptor signaling pathway (**Figure 1E**). To sum up, the result of functional enrichment analysis suggested that after immunization with inactivated vaccine of *A. salmonicida*, lots of the DEGs were enriched in a variety of immune-related signaling pathways.

### 3.2 Analysis of whole genome bisulfite sequencing

The inactivated vaccine of *A. salmonicida* was injected into turbot to induce the changes of methylation pattern, which were investigated by WGBS technology. The raw data of each sequenced sample was 120 million. After sequencing and quality control, the average clean ratio (clean reads/raw reads) in the AsVSm and NVSm samples was 97.48% and 97.24%, respectively. The Q30 value of each sequenced sample was higher than 89.61%, indicating that the sequencing result was credible and subsequent bioinformatics analysis could be carried out. BS Conversion Rate measures the success rate of bisulfite treatment of samples, and the results show that the conversion rate of each sample is above 99.7% (**Table 4**). The clean reads of six samples (AsVSm1-3, NVSm1-3) were compared to the reference genome (**Table 5**), and the unique mapping ratio were between 77.79% and 80.15%, and the duplication rates were below 17.96%, indicating that the sequencing has high quality and reliability. At the genome-wide level, the proportion of methylated C sites (mC) in different sequence environments (CG, CHG, CHH) was less different in both the AsVSm group and NVSm group, in which the average percentage of mC, mCG, mCHG and mCHH were 11.33%, 85.63%, 0.16% and 0.15% in NVSm group and were 11.07%, 85.67%, 0.20%, 0.17% in AsVSm group, respectively. (**Table 6**). Besides, the proportion of methylated C sites (mCG) in the mC sequence environment, which the average percentage of mCG was 98.54% in NVSm group and 98.76% in AsVSm group, was also the highest among the four sequence environments. (**Table 7**). These results showed that the methylation in turbot kidney tissue was mainly concentrated in the CG sequence environment, and more than 85.5% of the CG in genome-wide level and more than 98.5% of the CG in mC sequence environment was modified by methylation.

**Table 4:** Output Data quality of WGBS.

Sample name	Raw Reads	Clean Reads	Q30(%)	GC Content(%)	BS Conversion Rate(%)
NVSm_1	120000000	116381500	89.61	24.13	99.739
NVSm_2	120000000	116928214	90.1	23.5	99.755
NVSm_3	120000000	116755983	89.65	23.68	99.768
AsVSm_1	120000000	116236185	90.96	23.78	99.729
AsVSm_2	120000000	117169760	91.46	23.69	99.731
AsVSm_3	120000000	117543645	91.64	23.94	99.743

**Table 5:** Ratio of reads to the reference genome in WGBS.

Samples	Total reads	Mapped reads	Mapping rate(%)	Duplication rate(%)
NVSm_1	116381500	90533168	77.79	14.7
NVSm_2	116928214	91964040	78.65	10
NVSm_3	116755983	91373232	78.26	10.78
AsVSm_1	116236185	92128800	79.26	14.39
AsVSm_2	117169760	92610978	79.04	11.4
AsVSm_3	117543645	94211231	80.15	17.96

**Table 6:** Genome-wide methylation level.

Samples	mC percent(%)	mCpG percent(%)	mCHG percent(%)	mCHH percent(%)
NVSm1	11.05%	85.71%	0.17%	0.15%
NVSm2	11.01%	85.5%	0.15%	0.14%
NVSm3	11.04%	85.68%	0.16%	0.15%
AsVSm1	11.05%	85.64%	0.17%	0.16%
AsVSm2	11.08%	85.69%	0.21%	0.18%
AsVSm3	11.07%	85.68%	0.21%	0.18%

**Table 7:** The number and percentage distribution of mC.

Samples	mC Quantity and Ratio	mCG Quantity and Ratio	mCHG Quantity and Ratio	mCHH Quantity and Ratio
AsVSm1	24553255(100%)	24227051(98.67%)	87507(0.35%)	238697(0.97%)
AsVSm2	24618989(100%)	24241083(98.46%)	105825(0.42%)	272081(1.1%)
AsVSm3	24607157(100%)	24239064(98.5%)	103954(0.42%)	264139(1.07%)
NVSm1	24557640(100%)	24246585(98.73%)	83991(0.34%)	227064(0.92%)
NVSm2	24479029(100%)	24187260(98.8%)	78507(0.32%)	213262(0.87%)
NVSm3	24541005(100%)	24238210(98.76%)	81627(0.33%)	221168(0.9%)

**Figure 2:**
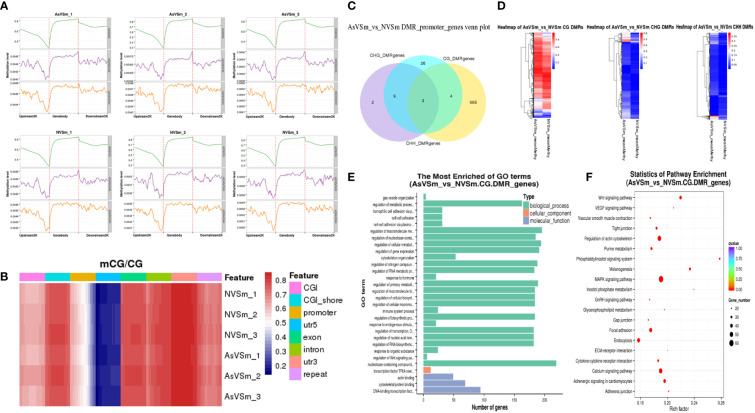
Screening of DMR and related genes by whole genome bisulfite sequencing. **(A)** The distribution of methylation level of samples at 2K upstream and downstream of gene region. **(B)** The results of thermographic analysis of methylation level of gene functional region in mCG sequence environment. **(C)** The number of intersections or unique gene sets of DMGs anchoring promoter regions in different sequence contexts (CG, CHG, CHH). **(D)** Clustering heatmap of DMR methylation levels in different sequence contexts (CG, CHG, CHH). **(E)** Go enrichment analysis of DMGs in the context of CG sequence. **(F)** The top 20 pathways significantly enriched for DMGs in the context of CG sequences.

The distribution of methylation levels in the upstream and downstream of regions was shown in **Figure 2A**. The highest average methylation level was shown in the CG sequence environment. In detail, the upstream2k regions were down-methylated compared to the of gene body regions, in which the methylation level gradually declined to a minimum at the transcription start site (TSS). Although the levels of CHG and CHH methylation were low in genome-wide level, the CHG and CHH methylation levels similar to mCG with consistent trends. Heat map analysis were used for more specific location analysis of the mCG sequence environment (**Figure 2B****)**. The result showed that CG methylation levels were lowest in the regions of promoter and 5’UTR around TSS, which are similar results as for **Figure 2A**.

DSS software was used to identify differentially methylated domains. A total of 8,149 DMRs were obtained in in the AsVSm and NVSm groups, including 4,377 hypermethylated domains (AsVSm/NVSm) and 3,772 hypomethylated domains (AsVSm/NVSm). The promoter is a DNA sequence that RNA polymerase recognized, bound and transcribed, which is generally located upstream of the TSS. Therefore, the analysis of the DMRs in the promoter region is very important to explore the regulation of DNA methylation on gene expression. The statistics of DMRs related genes anchored in the promoter regions were performed, and the results were shown in **Figure 2C**. The DMRs in the promoter region mainly exist in the environment of CG sequence, and among them, there were 665 DMGs that were unique in CG sequence. This phenomenon indicated that in the promoter region, the occurrence of DNA methylation was mainly concentrated in the environment of the CG sequence. Heat map analysis showed the differences between DMGs among different groups (**Figure 2D****)**. In the CG sequence environment, the DMR methylation levels of the AsVSm group and the NVSm group were significantly different. In the CHG and CHH sequence environment, although the comparison between the AsVSm group and the NVSm group showed significant differences in some regions, due to the low overall methylation level, we mainly focused on the CG sequence environment for subsequent analysis.

GO and KEGG enrichment analysis of DMRs were carried out. The DMGs were most significantly enriched in biological processes, including immune system process and regulation of Wnt signaling (**Figure 2E**). Besides, The DMRs were significant enriched in the immune-related signaling pathways, such as MAPK signaling pathway, Wnt pathway, Focal adhesion, Adhesion junction (**Figure 2F**). The analysis results confirmed that turbot immunized with inactivated vaccine changed the function of many immune related genes by altering methylation level, which might played a role in the immune process by affecting the gene expression level.

### 3.3 Association analysis between WGBS and transcriptome sequencing

**Figure 3:**
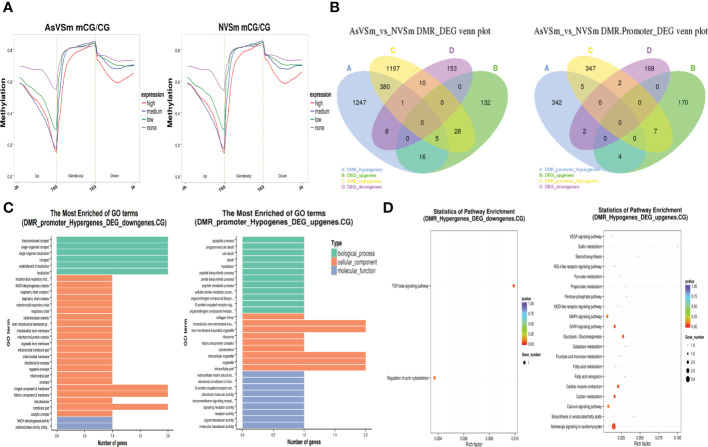
Regulation of methylation modification on gene expression level. **(A)** Distribution of gene expression level and methylation level in CG sequence environments. **(B)** Statistics of overlapping genes between DMGs and DEGs. **(C)** GO enrichment analysis of overlapping gene in promoter region CG sequence environment. **(D)** Significantly enriched pathway statistics for methylation negatively regulated genes.

The expression level of DEGs was divided into four levels: none, low, medium, and high, and their corresponding methylation level in the CG sequence environment was calculated. The results showed that DEGs in the AsVSm group and the NVSm group had similar DNA methylation patterns. In the environment of CG sequence, there was a significant negative correlation between the methylation level and the expression level of DEGs, and the negative regulatory was more significant with the closer distance to the TSS site (**Figure 3A**). In a word, the expression of hypomethylated genes was increased and the expression of hypermethylated genes was decreased. The results of combined analysis of DMGs and DEGs were shown in **Figure 3B**. There were a total of 42 negative regulatory genes located in the gene body region, in which 9 genes were hypermethylated with down-regulated expression, 33 genes were hypomethylated with up-regulated expression. And there were a total of 9 negative regulatory genes located in the promoter region, in which 2 genes were hypermethylated with down-regulated expression, and 7 genes were hypomethylated with up-regulated expression. We focused on the overlapping genes in the promoter region for GO enrichment analysis, the results were shown in **Figure 3C**. The hypermethylated genes with lower expression were mainly enriched in terms related to transport and localization in biological processes, membrane part in cellular components, and enzymatic activity in molecular functions. The hypomethylated genes with higher expression were mainly enriched in terms related to cell death in biological processes, organelles in cellular components, and transmembrane transport in molecular functions. Besides, GO enrichment analysis found that there were only 5 hypomethylation-high expression genes in the promoter region, including Eosinophil peroxidase-like (*EPX-Like*), Angiopoietin-related protein 5-like, C5a anaphylatoxin chemotactic receptor 1-like (*C5ar1-Like*), Arginase-2 mitochondrial, Hypothetical protein. KEGG enrichment analysis showed that hypermethylated genes with lower expression and hypomethylated genes with higher expression were significantly enriched in different signaling pathways (**Figure 3D**). In particular, many hypomethylated genes with higher expression were enriched in immune-related pathways, such as TGF-beta signaling pathway, MAPK signaling pathway and NOD-like receptor signaling pathway. Therefore, further study will focus on hypomethylated genes with higher expression to study their immune regulatory mechanisms.

### 3.4 DNA methylation negatively regulates gene expression

**Figure 4:**
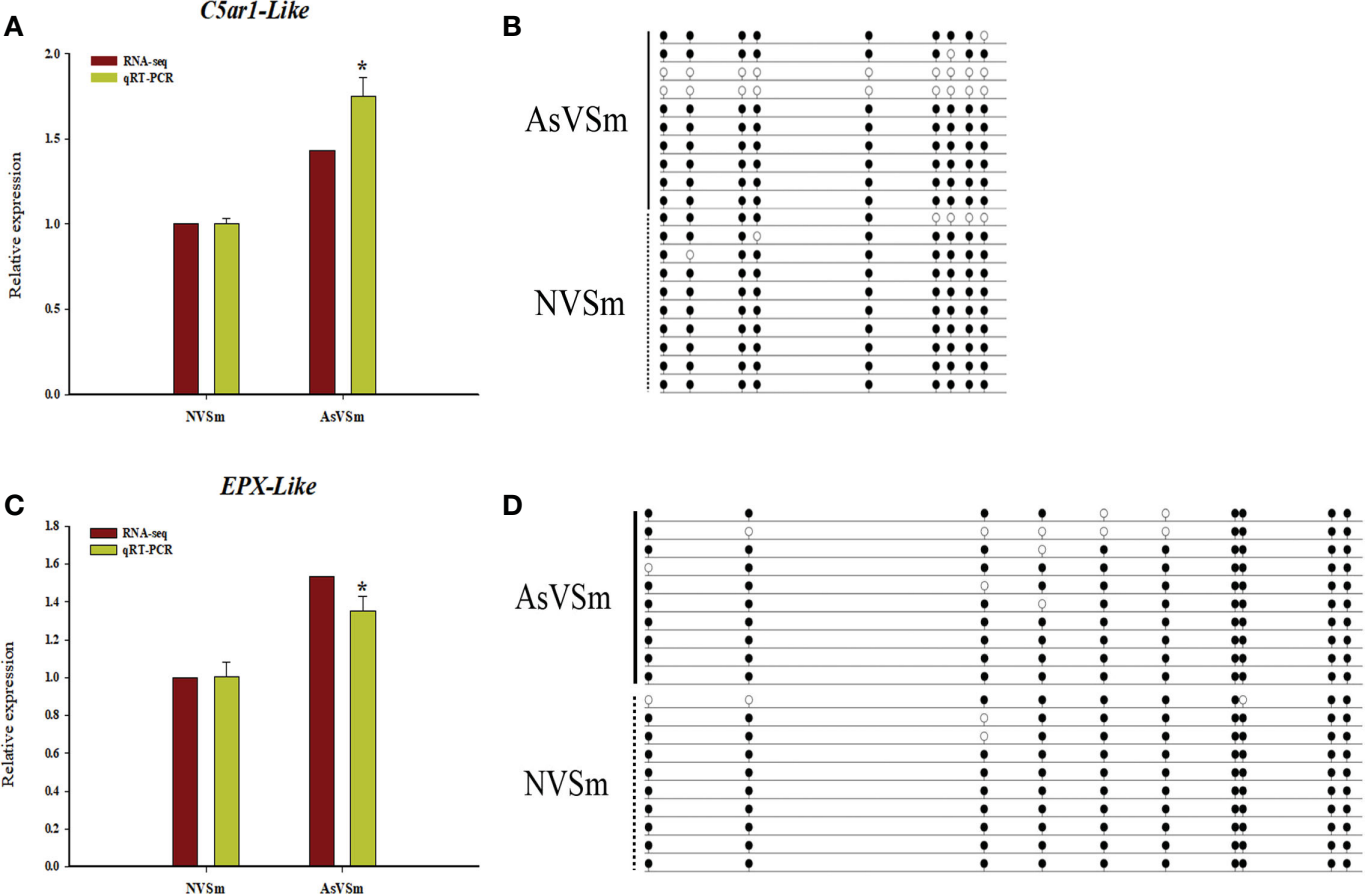
DNA methylation negatively regulated gene expression. **(A)** The relative expression levels of *C5ar1-Like* gene in AsVSm group and NVSm group. **(B)** The average methylation level of the promoter regions of the *C5ar1-Like* gene in AsVSm group and NVSm group. **(C)** The relative expression levels of *EPX-Like* gene in AsVSm group and NVSm group. **(D)** The average methylation level of the promoter regions of the *EPX-Like* gene in AsVSm group and NVSm group. *p<0.05.

According to combined analysis, we selected two immune-related genes, *C5ar1-Like* and *EPX-Like*, with lower methylation in the promoter region and higher expression level as candidate genes to verify the reliability of WGBS result and transcriptomic results. Real-time PCR was used to detect the relative expression levels of *C5ar1-Like* and *EPX-Like* genes in AsVSm group and NVSm group. Experimental results showed that the expression level of *C5ar1-Lik*e and *EPX-Like* genes in AsVSm group was significantly higher than that in NVSm group (*P <*0.05) (**Figure 4A, C****)**. The verification results proved that the expression level of *C5ar1-Like* and *EPX-Like* genes was consistent with the transcriptome sequencing. BSP sequencing results showed that the average methylation level of the promoter regions of the *C5ar1-Like* and *EPX-Like* genes in the AsVSm group was significantly lower than that in the NVSm group, indicating that the BSP sequencing results were consistent with the WGBS sequencing results (**Figure 4B, D****)**. Methylation and expression level verification experiments demonstrated that the expression level of *C5ar1-Like* and *EPX-Like* genes was increased, and the methylation level was decreased after the turbot was injected with the inactivated vaccine of *A. salmonicida*.

### 3.5 The mechanism of DNA methylation inhibited immune-related gene expression

**Figure 5:**
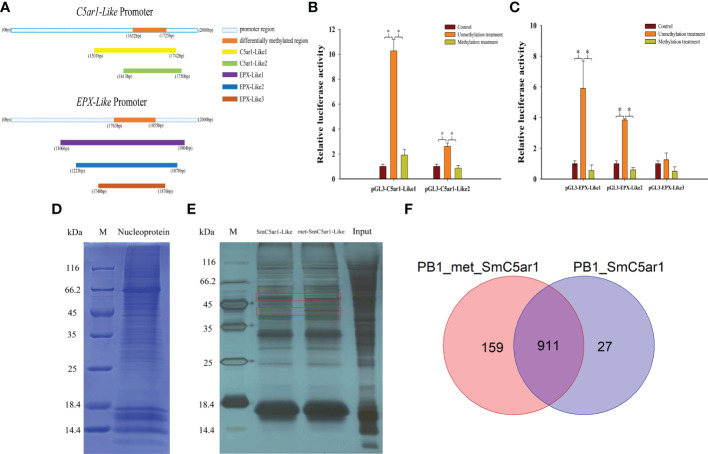
The mechanism of DNA methylation inhibited immune-related gene expression. **(A)** Amplified fragments from *C5ar1-Like* and *EPX-Like* promoter regions. The different colors represent different gene fragments. **(B)** Effects of DNA methylation modifications on fluorescent activity of *C5ar1-Like* promoter regions. **(C)** Effects of DNA methylation modifications on fluorescent activity of *EPX-Like* promoter regions.*Representative significant difference (*P* < 0.05). **(D)** Coomassie blue staining of HEK293T nuclear proteins. **(E)** Silver staining of DNA pull down enriched proteins. The red box shows the different bands between the met-SmC5ar1-Like and the SmC5ar1-Like. **(F)** Total number of the met-SmC5ar1-Like and the SmC5ar1-Like interacting proteins identified by MS.

We constructed a series of dual luciferase reporter gene recombinant plasmids (pGL3-C5ar1-Like1, pGL3-C5ar1-Like2 and pGL3-EPX-Like1, pGL3-EPX-Like2, pGL3-EPX-Like3) around the DMR in the promoter regions of *C5ar1-Like* and *EPX-Like* genes to perform methylation modification *in vitro* (**Figure 5A**). The results of the dual luciferase report experiment showed that the effects of C5ar1-Like and EPX-Like promoter methylation on gene expression were similar to the above results (**Figure 5B,C**). One side, compared with the pGL3-basic empty vector (Control), the relative luciferase activity of the unmethylated recombinant plasmid (Unmethylation treatment) was significantly increased, proving that the recombinant promoter sequence has transcriptional activity. Other side, compared with the unmethylated recombinant plasmid, the relative luciferase activity of the methylated recombinant plasmid (Methylation treatment) was significantly reduced. In detail, the transcriptional activity of C5ar1-like2 was lower than that of C5ar1-like1, but the same trend was observed in C5ar1-like1 and C5ar1-like2, which indicates that methylation could effectively inhibit the transcriptional activity of both. Similarly, the transcriptional activity of EPX-Like2 was lower than that of EPX-Like2, but the same trend was observed in EPX-Like1 and EPX-Like2, which indicates that methylation could also effectively inhibit the transcriptional activity of both. In particular, no significant changes in luciferase activity of EPX-Like3 promoter were observed in unmethylation treatment group compared with control group, indicating EPX-Like3 transcription inactivation. Therefore, there was also no significant change in fluorescence activity of EPX-Like3 promoter in methylation treatment group compared with control group. These results showed that the transcriptional activity of EPX-Like3 promoter were not impacted by the methylated modification because of EPX-Like3 transcription inactivation. According to the experimental results, we speculated that the DNA methylation directly modified the promoter of *C5ar1-Like* and *EPX-Like* genes and inhibited the transcriptional activity of the promoters, thereby inhibited the expression of genes.

The interaction proteins with the probes of met-SmC5ar1-Like and the SmC5ar1-Like were screened by DNA pull down. The nuclear protein of HEK293T cells was extracted for sodium dodecyl sulfate Polycrylamide gel electrophoresis (SDS-PAGE) (**Figure 5D**) and DNA pull down (**Figure 5E**). Then, the interacting proteins with the met-SmC5ar1-Like and the SmC5ar1-Like were verified by WB. The silver staining results of enriched proteins showed that there were obvious different bands between the met-SmC5ar1-Like and the SmC5ar1-Like, which indicated that the interacting proteins with the met-SmC5ar1-Like had obvious changes compared with the SmC5ar1-Like (**Figure 5E**). Then, all ofthe interacting proteins with the met-SmC5ar1-Like and the SmC5ar1-Like were identified by MS. Using Homo sapiens database as a reference database, and the Proteome Discoverer 2.4 software was used to retrieve the database to determine the properties of interacting proteins. MS results showed that a total of 1,097 interacting proteins were identified, including 911 common interacting proteins, 159 the met-SmC5ar1-Like specific binding proteins and 27 the SmC5ar1-Like specific binding proteins (**Figure 5F**). The relevant information of met-SmC5ar1-Like and SmC5ar1-Like specific binding proteins can be found in the **Supplementary Table S1** and **Supplementary Table S2**, respectively. The result indicated that DNA methylation effected binding of the interaction proteins to the promoter region of SmC5ar1-Like and further influenced the gene expression and immune responses.

## 4 Discussion

DNA methylation was generally considered to have a regulatory effect on gene expression. Therefore, the combined analysis of WGBS and transcriptome sequencing has been widely conducted in the related research of mammal (18, 19). Besides, this combined analysis also has been widely reported in aquatic animals. In growth and development, RNA-seq and WGBS sequencing results association analysis was conducted in common carp, and the results showed that the expression levels of PUFA-related genes in muscle tissue were significantly correlated with their methylation status (20). However, this research model has hardly been applied in the immune response of turbot. In this study, the combined analysis of WGBS and transcriptome sequencing was used for the first time to explore the DNA methylation pattern and gene expression pattern of in kidney tissue of control and inactivated vaccine-infected turbot. We found no significant changes in the genome-wide DNA methylation pattern in the kidney tissue of turbot after immunization with inactivated vaccine of *A. salmonicida*. However, analysis of the WGBS results revealed a total of 4,377 hypermethylated domains and 3,772 hypomethylated domains in the AsVSm group. This phenomenon reflected that the change of DNA methylation pattern of AsVSm group samples was not the change of overall DNA methylation level, but the change of DNA methylation level of a large number of genes. In general, DNA methylation was thought to occur universally in vertebrates, and CG sites were hypermethylated in genome-wide, also in turbot. DNA methylation was rare in the context of CHG and CHH sequences and was only present in some specialized tissues, such as the brain and embryonic stem cells (21, 22), speculating that CHG and CHH methylation patterns may play a role in special tissues. Our data showed that in the AsVSm group and the NVSm group, methylated C sites accounted for about 11% of the total C sites, mCG accounted for about 98% of the total methylated C sites, and mCG accounted for about 85% of the total CG sites. Combined with the results of gene methylation level distribution, it was not difficult to find that mCG was mainly distributed in the genebody and its downstream 2K region, and the methylation level was low in the promoter region, especially in the region near the TSS. Obviously, most of the CG sites near TSS were not methylated. This situation might be due to the distribution of many CpG islands (CGI) with high CpG sequence density in the promoter region. According to statistics, about 70% of vertebrate annotated gene promoters are related to CGI (23, 24) and CG sites in CGI are generally in a non-methylated state, while CG sites located outside CGI are more easily modified by methylation, so the methylation level of promoter regions was significantly lower than that of other gene functional elements (25). When CGI undergo methylation modification, the methylation level of promoter region changes and generally accompanied by the inhibition of gene transcription, resulting in gene silencing (26, 27). The distribution of upstream and downstream methylation levels of genes indicated high methylation levels in this genomic region, which is common in mammalian, especially in aquatic animals, and negatively regulated the level of gene expression (28, 29). These results indicated that the spontaneous DNA methylation pattern in turbot was similar to other species, indicating that the DNA methylation patterns among vertebrates are relatively conserved to a certain extent.

In addition, the injection of inactivated vaccine of *A. salmonicida* changed the methylation level of several genes in turbot kidney tissue. It was found from the WGBS results that many DMGs were significantly enriched in multiple immune-related pathways, such as MAPK signaling pathway, Wnt signaling pathway. Among them, MAPK signaling pathway plays a key role in apoptosis and the interaction between bacteria and host in cancer and immune system (29, 30). At the same time, a large number of immune-related DEGs were screened and analyzed from the transcriptome sequencing results. Multiple immune related DEGs, such as Toll-like receptor 7 (TLR7), transcription factor activator protein 1 (AP-1), Signal transducer and activator of transcription (STAT), Tumor necrosis factor receptor superfamily member 5 (TNFSF5), Mitogen-activated protein kinase 14A (MAPK14A) and Anthrax toxin receptor 1 (Antxr1), were significantly enriched in multiple immune-related pathways, including the two most significant pathways, Toll-like receptor signaling pathway and NOD-like receptor signaling pathway. Toll-like receptor (TLR) family was an important pathogen-related molecular pattern recognition receptor, which could activate downstream NF-κB and mediated the expression of transcription factor AP-1 to induce the expression of inflammatory cytokines such as tumor necrosis factor, interleukins, and interferon, triggering inflammatory response and playing an important role in immune response (31–33). NOD-like receptor family could recognized pathogen-mediated damage signals and activated NF-κB and MAPK signaling pathways to participate in inflammatory and immune responses (34). In general, after immunization with inactivated vaccine, the changes of methylation level modified expression level of immune-related genes, and finally triggered the immune response in turbot.

Specific relationships between inactivated vaccines-mediated DNA methylation and immune responses were demonstrated by combined analysis of WGBS and RNA-seq. Two immune-related negative regulatory genes, *C5ar1-Like* and *EPX-Like*, were screened from the immune-related genes in the combined analysis of DMRs-DEGs. C5a anaphylatoxin as a potent soluble mediator triggered chemotactic inflammation and the innate immune response *via* effecting inflammatory cell chemotaxis, chemokine release, phagocytosis as well as recruiting neutrophils and macrophages (35, 36). As a specific receptor of C5a, C5ar could cause pro-inflammatory activation after specific binding with C5a in grass carp and zebrafish (37, 38). Studies have shown that in the vaccinated rainbow trout, the expression of C5ar gene was up-regulated and enhance the binding of C5ar to C5a, inducing chemotactic response in granulocytes and immune response in response to *Yersinia ruckeri* (39). Eosinophil peroxidase (EPX) as a major cationic protein found in immune cell of human and mouse was highly conserved (40). In fish, EPX has been reported that was a vital component of the immune system of starry flounder (*Platichthys stellatus*), showing significant antibacterial and antiviral activity against *Streptococcus parauberis* and viral *haemorrhagic septicaemia* virus infection (41). In this study, the methylation level of *C5ar1-Like* gene and *EPX-Like* gene in the AsVSm group were significantly lower than that in the NVSm group, and the expression level in the AsVSm group was significantly higher than that in the NVSm group. Therefore, we inferred that DNA methylation was very likely to negatively regulate the expression of the *C5aR1-Like* and *EPX-Like* gene. In order to accurately verify the methylation modification site in the promoter region of the *C5aR1-Like* and *EPX-Like* gene, the dual-luciferase reporter assay was conducted. The results showed that methylation modification significantly inhibited the fluorescent activity of *C5aR1-Like* and *EPX-Like* in the promoter region. Meanwhile, the methylation modification of the *EPX-like* promoter region significantly reduced its expression level. These finding confirmed that DNA methylation directly modified the promoter of *C5ar1-Like* and *EPX-Like* genes and might inhibit the transcriptional activity of the promoters, thereby inhibited the expression of genes.

In order to prove that DNA methylation modification regulated gene expression by affecting transcription factor binding, the differential binding proteins of *C5ar1-Like* gene promoter were identified and screened by DNA pull down and MS. We found that compared with met-SmC5ar1-Like, SmC5ar1-Like has 27 unique interacting proteins, indicating that these proteins were unable to bind to the *C5ar1-Like* promoter after DNA methylation modification. These specific binding transcription factors included transcription-related transcription factors, translation-related transcription factors, signaling-related transcription factors, and also included some immune-related transcription factors such as eukaryotic translation initiation factor 2B (eIF-2B), talin-1 (TLN1), immunoglobulin heavy constant mu (IGHM). Among them, eIF-2B could affect the initiation phase of mRNA translation and was essential for modulating white matter disease in zebrafish (42). TLN1 could induce heart disease *via* suppressing the PI3K/AKT signaling pathway in zebrafish (43). The overexpression of IGHM could activate the immune response during bacterial and parasite infection in *Labrus bergylta* (44). The absence of these transcription factors greatly affected the immune response. Therefore, DNA methylation effected binding of transcription factors to the gene promoter regions and further influenced the transcription factors to regulate immune response.

In conclusion, a number of DNA methylation and the gene expression in turbot kidney tissue was changed after vaccination with inactivated vaccine of *A. salmonicida*. In addition, the result of dual luciferase reporting experiment and DNA pull down confirmed that DNA methylation modification in gene promoter region can affect gene expression *via* inhibiting the binding of transcription factors with gene promoter region. Our results reveal that inactivated vaccine affected the level of DNA methylation and gene expression in turbot kidney tissue, which explored the potential application of DNA methylation in turbot resistance breeding, and provided a new idea for genetic improvement of turbot.

## Data availability statement

The datasets presented in this study can be found in online repositories. The names of the repository/repositories and accession number(s) can be found below: https://doi.org/10.6084/m9.figshare.21821160.v1.

## Ethics statement

All fish experimental procedures were carried out in accordance with the National Institutes of Health’s Guide for the Care and Use of Laboratory Animals, as implemented by Qingdao Agricultural University. The convention has been ratified by the IACUC Committee on the Ethics of Animal Experiments at Qingdao Agricultural University (Institutional Animal Care and Use Committee).

## Author contributions

YX contributed to conception and design of the study. YL analyzed the data, and drafted manuscript. LS provided helpful discussions, XL and HG organized the database. SZ commented on the manuscript. All authors contributed to manuscript revision, read, and approved the submitted version.

## References

[1] Shi G Feng J Jian LY Fan XY. DNA Hypomethylation promotes learning and memory recovery in a rat model of cerebral Ischemia/Reperfusion injury. Neural Regeneration Res (2023) 18(4):863–8. doi: 10.4103/1673-5374.353494 PMC9700107 36204855

[2] Zhang L Lu Q Chang C. Epigenetics in health and disease. Adv Exp Med Biol (2020) 1253:3–55. doi: 10.1007/978-981-15-3449-2_1 32445090

[3] Shang X Wan Q Su J Su J. DNA Methylation of cirig-I gene notably relates to the resistance against gcrv and negatively-regulates mrna expression in grass carp, ctenopharyngodon idella. Immunobiology (2016) 221(1):23–30. doi: 10.1016/j.imbio.2015.08.006 26314762

[4] Hu Q Ao Q Tan Y Gan X Luo Y Zhu J. Genome-wide DNA methylation and rna analysis reveal potential mechanism of resistance to streptococcus agalactiae in gift strain of Nile tilapia (*Oreochromis niloticus* ). J Immunol (Baltimore Md 1950) (2020) 204(12):3182–90. doi: 10.4049/jimmunol.1901496 PMC7276942 32332111

[5] Pereiro P Figueras A Novoa B. Turbot (*Scophthalmus maximus*) vs. Vhsv (Viral Hemorrhagic Septicemia Virus): A Review. Front Physiol (2016) 7:192. doi: 10.3389/fphys.2016.00192 27303308 PMC4880558

[6] He X Song X Cao H Zhou Q Zhang J Yue H. Glaesserella parasuis induces il-17 production might through pkc-Erk/Mapk and Iκb/Nf-κb signaling pathways. Veterinary Microbiol (2022) 273:109521. doi: 10.1016/j.vetmic.2022.109521 35932516

[7] Liu X Wang B Gao C Xue T Liu Z Su B. Characterization and the potential immune role of class a scavenger receptor member 4 (Scara4) in bacterial infection in turbot (*Scophthalmus maximus l.*). Fish shellfish Immunol (2022) 120:590–8. doi: 10.1016/j.fsi.2021.12.041 34965442

[8] Huimin; G Yuanyuan; D Hao; W Xujia; Z Yunji; X Shun Z. Preparation of *Aeromonas salmonicida* inactivated vaccine and test of its immunological efficacy in *Scophthalmus maximus* . J Fisheries China (2021) 45(9):1574–83. doi: 10.11964/jfc.20210612932

[9] Ansell BRE Bahlo M Bannister S Kim B Domínguez-Andrés J Vlahos A. Neonatal bcg vaccination is associated with a long-term DNA methylation signature in circulating monocytes. Sci Adv (2022) 8(31):eabn4002. doi: 10.1126/sciadv.abn4002 35930640 PMC9355358

[10] Fairfax BP Martinon-Torres F Pischedda S O'Connor D Pollard AJ Salas A. Changes in epigenetic profiles throughout early childhood and their relationship to the response to pneumococcal vaccination. Clin Epigenet (2021) 13-25(1):29. doi: 10.1186/s13148-021-01012-w PMC7860179 33541404

[11] Al-Quraishy S Dkhil MA Abdel-Baki AS Ghanjati F Erichsen L Santourlidis S. Protective vaccination and blood-stage malaria modify DNA methylation of gene promoters in the liver of Balb/C mice. Parasitol Res (2017) 116(5):1463–77. doi: 10.1007/s00436-017-5423-0 28315013

[12] Farrell C Thompson M. Bisulfite bolt: A bisulfite sequencing analysis platform. GigaScience (2021) 10(5):1–8. doi: 10.1093/gigascience/giab033 PMC8106542 33966074

[13] Langmead B Salzberg SL. Fast gapped-read alignment with bowtie 2. Nat Methods (2012) 9(4):357–9. doi: 10.1038/nmeth.1923 PMC3322381 22388286

[14] Lister R Mukamel EA Nery JR Urich M Puddifoot CA Johnson ND. Global epigenomic reconfiguration during mammalian brain development. Sci (New York NY) (2013) 341(6146):1237905. doi: 10.1126/science.1237905 PMC3785061 23828890

[15] Park Y Wu H. Differential methylation analysis for bs-seq data under general experimental design. Bioinf (Oxford England) (2016) 32(10):1446–53. doi: 10.1093/bioinformatics/btw026 PMC12157722 26819470

[16] Young MD Wakefield MJ Smyth GK Oshlack A. Gene ontology analysis for rna-seq: Accounting for selection bias. Genome Biol (2010) 11(2):R14–26. doi: 10.1186/gb-2010-11-2-r14 PMC2872874 20132535

[17] Wang LJ Qiu BQ Yuan MM Zou HX Gong CW Huang H. Identification and validation of dilated cardiomyopathy-related genes *Via* bioinformatics analysis. Int J Gen Med (2022) 15:3663–76. doi: 10.2147/ijgm.s350954 PMC8994656 35411175

[18] Zhang D Wu S Zhang X Ren S Tang Z Gao F. Coordinated transcriptional and post-transcriptional epigenetic regulation during skeletal muscle development and growth in pigs. J Anim Sci Biotechnol (2022) 13(1):146–60. doi: 10.1186/s40104-022-00791-3 PMC9714148 36457054

[19] Ju Z Jiang Q Wang J Wang X Yang C Sun Y. Genome-wide methylation and transcriptome of blood neutrophils reveal the roles of DNA methylation in affecting transcription of protein-coding genes and mirnas in e. Coli-Infected Mastitis Cows. BMC Genomics (2020) 21(1):102–16. doi: 10.1186/s12864-020-6526-z PMC6993440 32000686

[20] Zhang H Xu P Jiang Y Zhao Z Feng J Tai R. Genomic, transcriptomic, and epigenomic features differentiate genes that are relevant for muscular polyunsaturated fatty acids in the common carp. Front Genet (2019) 10:217. doi: 10.3389/fgene.2019.00217 30930941 PMC6428711

[21] Lister R Pelizzola M Dowen RH Hawkins RD Hon G Tonti-Filippini J. Human DNA methylomes at base resolution show widespread epigenomic differences. Nature (2009) 462(7271):315–22. doi: 10.1038/nature08514 PMC2857523 19829295

[22] Feng S Cokus SJ Zhang X Chen PY Bostick M Goll MG. Conservation and divergence of methylation patterning in plants and animals. Proc Natl Acad Sci United States America (2010) 107(19):8689–94. doi: 10.1073/pnas.1002720107 PMC2889301 20395551

[23] Saxonov S Berg P. Brutlag DL A genome-wide analysis of cpg dinucleotides in the human genome distinguishes two distinct classes of promoters. Proc Natl Acad Sci United States America (2006) 103(5):1412–7. doi: 10.1073/pnas.0510310103 PMC1345710 16432200

[24] Ma Z Wang Y Quan Y Wang Z Liu Y Ding Z. Maternal obesity alters methylation level of cytosine in cpg island for epigenetic inheritance in fetal umbilical cord blood. Hum Genomics (2022) 16(1):34–45. doi: 10.1186/s40246-022-00410-2 36045397 PMC9429776

[25] Cardenas H Fang F Jiang G Perkins SM Zhang C Emerson RE. Methylomic signatures of high grade serous ovarian cancer. Epigenetics (2021) 16(11):1201–16. doi: 10.1080/15592294.2020.1853402 PMC8813084 33289590

[26] Zou Z Zhang Y Huang Y Wang J Min W Xiang M. Integrated genome-wide methylation and expression analyses provide predictors of diagnosis and early response to antidepressant in panic disorder. J Affect Disord (2023) 322:146–55. doi: 10.1016/j.jad.2022.10.049 36356898

[27] Xu J Zhang W Zhong S Xie X Che H Si W. Microcystin-Leucine-Arginine affects brain gene expression programs and behaviors of offspring through paternal epigenetic information. Sci total Environ (2023) 857(Pt 1):159032. doi: 10.1016/j.scitotenv.2022.159032 36167133

[28] Yang L Liu L Cheng J Wu Z Bao W Wu S. Association analysis of DNA methylation and the Tissue/Developmental expression of the Fut3 gene in meishan pigs. Gene (2023) 851:147016. doi: 10.1016/j.gene.2022.147016 36374642

[29] Liu Z Zhou T Gao D. Genetic and epigenetic regulation of growth, reproduction, disease resistance and stress responses in aquaculture. Front Genet (2022) 13:994471. doi: 10.3389/fgene.2022.994471 36406125 PMC9666392

[30] Yin C Gu J Gu D Wang Z Ji R Jiao X. The salmonella T3ss1 effector ipaj is regulated by itra and inhibits the mapk signaling pathway. PloS Pathog (2022) 18(12):e1011005. doi: 10.1371/journal.ppat.1011005 36477497 PMC9728880

[31] O'Neill LA Golenbock D Bowie AG. The history of toll-like receptors - redefining innate immunity. Nat Rev Immunol (2013) 13(6):453–60. doi: 10.1038/nri3446 23681101

[32] Kawai T Akira S. The role of pattern-recognition receptors in innate immunity: Update on toll-like receptors. Nat Immunol (2010) 11(5):373–84. doi: 10.1038/ni.1863 20404851

[33] DiDonato JA Mercurio F Karin M. Nf-κb and the link between inflammation and cancer. Immunol Rev (2012) 246(1):379–400. doi: 10.1111/j.1600-065X.2012.01099.x 22435567

[34] Cao X. Self-regulation and cross-regulation of pattern-recognition receptor signalling in health and disease. Nat Rev Immunol (2016) 16(1):35–50. doi: 10.1038/nri.2015.8 26711677

[35] Mateu-Borrás M González-Alsina A Doménech-Sánchez A Querol-García J Fernández FJ Vega MC. *Pseudomonas aeruginosa* adaptation in cystic fibrosis patients increases C5a levels and promotes neutrophil recruitment. Virulence (2022) 13(1):215–24. doi: 10.1080/21505594.2022.2028484 PMC8802900 35094639

[36] Roewe J Walachowski S Sharma A Berthiaume KA Reinhardt C Bosmann M. Bacterial polyphosphates induce Cxcl4 and synergize with complement anaphylatoxin C5a in lung injury. Front Immunol (2022) 13:980733. doi: 10.3389/fimmu.2022.980733 36405694 PMC9669059

[37] Li L Yang W Shen Y Xu X Li J. The evolutionary analysis of complement component C5 and the gene Co-expression network and putative interaction between C5a and C5a anaphylatoxin receptor (C5ar/Cd88) in human and two cyprinid fish. Dev Comp Immunol (2021) 116:103958. doi: 10.1016/j.dci.2020.103958 33290783

[38] Pandey MK Trivedi VS Magnusen AF Rani R Marsili L. Targeting the complement-sphingolipid system in covid-19 and gaucher diseases: Evidence for a new treatment strategy. Int J Mol Sci (2022) 23(22):103390. doi: 10.3390/ijms232214340 PMC9695449 36430817

[39] Raida MK Buchmann K. Bath vaccination of rainbow trout (*Oncorhynchus mykiss walbaum*) against *Yersinia ruckeri*: Effects of temperature on protection and gene expression. Vaccine (2008) 26(8):1050–62. doi: 10.1016/j.vaccine.2007.12.029 18237828

[40] Percopo CM Krumholz JO Fischer ER Kraemer LS Ma M Laky K. Impact of eosinophil-peroxidase (Epx) deficiency on eosinophil structure and function in mouse airways. J Leukocyte Biol (2019) 105(1):151–61. doi: 10.1002/jlb.3ab0318-090rr PMC6310085 30285291

[41] Choi KM Joo MS Kang G Woo WS Kim KH Jeong SH. First report of eosinophil peroxidase in starry flounder (*Platichthys stellatus*): Gene identification and gene expression profiling. Fish Shellfish Immunol (2021) 118:155–9. doi: 10.1016/j.fsi.2021.08.021 34461259

[42] Kang HC Kim CH Kim D Lee YR Choi TI Kim SH. Comparative proteome research in a zebrafish model for vanishing white matter disease. Int J Mol Sci (2021) 22(5):2707–19. doi: 10.3390/ijms22052707 PMC7962458 33800130

[43] Yu B Yao S Liu L Li H Zhu J Li M. The role of polypeptide Pdtln1 in suppression of Pi3k/Akt signaling causes cardiogenetic disorders in vitro and in vivo. Life Sci (2022) 289:120244. doi: 10.1016/j.lfs.2021.120244 34922940

[44] Zhou W Krogdahl Å Sæle Ø Chikwati E Løkka G Kortner TM. Digestive and immune functions in the intestine of wild ballan wrasse (*Labrus bergylta*). Comp Biochem Physiol Part A Mol Integr Physiol (2021) 260:111011. doi: 10.1016/j.cbpa.2021.111011 34174428

