# Supplementary material for: Immunity of turbot Induced by inactivated vaccine of Aeromonas salmonicida from the perspective of DNA methylation
Source: Front Immunol. 2023 Feb 7;14:1124322. doi: 10.3389/fimmu.2023.1124322 (PMC9945314; doi:10.3389/fimmu.2023.1124322)
Supplement: Supplementary file 1 [file Table_1.docx]

**Supplementary Table S1**. List of 159 the met-SmC5ar1-Like specific binding proteins.

| Met-SmC5ar1-Like specific binding proteins | Description |
| --- | --- |
| WDR1 | WD repeat-containing protein 1 |
| PSMD4 | 26S proteasome non-ATPase regulatory subunit 4 |
| RHOT2 | Mitochondrial Rho GTPase 2 |
| ARHGDIA | Rho GDP-dissociation inhibitor 1 |
| VTN | Vitronectin |
| ATP5F1 | ATP synthase F(0) complex subunit B1, mitochondrial |
| RPL8 | 60S ribosomal protein L8 |
| ACTR1A | Alpha-centractin |
| KARS | Lysine--tRNA ligase |
| RPL21 | 60S ribosomal protein L21 |
| ATP6V1A | V-type proton ATPase catalytic subunit A |
| HMGB1 | High mobility group protein B1 |
| PLRG1 | Pleiotropic regulator 1 |
| TOMM22 | Mitochondrial import receptor subunit TOM22 homolog |
| PYGL | Glycogen phosphorylase, liver form |
| KHDRBS3 | KH domain-containing, RNA-binding, signal transduction-associated protein 3 |
| TUBB6 | Tubulin beta-6 chain |
| NSUN2 | RNA cytosine C(5)-methyltransferase NSUN2 |
| HMGB3 | High mobility group protein B3 OS=Homo sapiens |
| RPS25 | 40S ribosomal protein S25 |
| EIF3K | Eukaryotic translation initiation factor 3 subunit K |
| ANP32E | Acidic leucine-rich nuclear phosphoprotein 32 family member E |
| NUP133 | Nuclear pore complex protein Nup133 |
| HNRNPUL2 | Heterogeneous nuclear ribonucleoprotein U-like protein 2 |
| ENY2 | Transcription and mRNA export factor ENY2 |
| BSG | Basigin |
| MAP4 | Microtubule-associated protein 4 |
| POLR2E | DNA-directed RNA polymerases I, II, and III subunit RPABC1 |
| LRRD1 | Leucine-rich repeat and death domain-containing protein 1 |
| HNRNPA0 | Heterogeneous nuclear ribonucleoprotein A0 |
| VRK1 | Serine/threonine-protein kinase VRK1 |
| COPS6 | COP9 signalosome complex subunit 6 |
| NUP210 | Nuclear pore membrane glycoprotein 210 |
| CAPRIN1 | Caprin-1 |
| PURA | Transcriptional activator protein Pur-alpha |
| DDX27 | Probable ATP-dependent RNA helicase DDX27 |
| PRRC2C | Protein PRRC2C |
| NT5DC1 | 5'-nucleotidase domain-containing protein 1 |
| RPS26 | 40S ribosomal protein S26 |
| NOP2 | Probable 28S rRNA (cytosine(4447)-C(5))-methyltransferase |
| MRE11 | Double-strand break repair protein MRE11 |
| RPL18A | 60S ribosomal protein L18a |
| FAM98B | Protein FAM98B |
| DPM1 | Dolichol-phosphate mannosyltransferase subunit 1 |
| NME4 | Nucleoside diphosphate kinase, mitochondrial |
| RPL35 | 60S ribosomal protein L35 |
| CHCHD3 | MICOS complex subunit MIC19 |
| COPS2 | COP9 signalosome complex subunit 2 |
| CYC1 | Cytochrome c1, heme protein, mitochondrial |
| ACOT9 | Acyl-coenzyme A thioesterase 9, mitochondrial |
| RNH1 | Ribonuclease inhibitor |
| BYSL | Bystin |
| TP53 | Cellular tumor antigen p53 |
| SERPINF2 | Alpha-2-antiplasmin |
| TES | Testin |
| BZW2 | Basic leucine zipper and W2 domain-containing protein 2 |
| FAM98A | Protein FAM98A |
| USP39 | U4/U6.U5 tri-snRNP-associated protein 2 |
| PLAA | Phospholipase A-2-activating protein |
| PGM1 | Phosphoglucomutase-1 |
| CYB5R3 | NADH-cytochrome b5 reductase 3 |
| CRNN | Cornulin |
| NPLOC4 | Nuclear protein localization protein 4 homolog |
| DAZAP1 | DAZ-associated protein 1 |
| SAFB | Scaffold attachment factor B1 |
| SART1 | U4/U6.U5 tri-snRNP-associated protein 1 |
| NAE1 | NEDD8-activating enzyme E1 regulatory subunit |
| CBX3 | Chromobox protein homolog 3 |
| DDX39A | ATP-dependent RNA helicase DDX39A |
| SCAMP3 | Secretory carrier-associated membrane protein 3 |
| PSMA5 | Proteasome subunit alpha type-5 |
| NIPSNAP1 | Protein NipSnap homolog 1 |
| SRP9; SRP9P1 | Signal recognition particle 9 kDa protein |
| MOGS | Mannosyl-oligosaccharide glucosidase |
| RAE1 | mRNA export factor |
| C21orf33 | Glutamine amidotransferase-like class 1 domain-containing protein 3B, mitochondrial |
| CGGBP1 | CGG triplet repeat-binding protein 1 |
| SF3B14; SF3B6 | Splicing factor 3B subunit 6 |
| KRT8 | Keratin, type II cytoskeletal 8 |
| ACOT1 | Acyl-coenzyme A thioesterase 1 |
| NOC2L | Nucleolar complex protein 2 homolog |
| CHORDC1 | Cysteine and histidine-rich domain-containing protein 1 |
| OXCT1 | Succinyl-CoA:3-ketoacid coenzyme A transferase 1, mitochondrial |
| SMC4 | Structural maintenance of chromosomes protein 4 |
| NT5DC2 | 5'-nucleotidase domain-containing protein 2 |
| MRPL11 | 39S ribosomal protein L11, mitochondrial |
| LRRC47 | Leucine-rich repeat-containing protein 47 |
| NHP2L1 | NHP2-like protein 1 |
| SH3GL1 | Endophilin-A2 |
| HNRNPUL1 | Heterogeneous nuclear ribonucleoprotein U-like protein 1 |
| SNRPD1 | Small nuclear ribonucleoprotein Sm D1 |
| MRPS14 | 28S ribosomal protein S14, mitochondrial |
| TCEA1 | Transcription elongation factor A protein 1 |
| UCHL5 | Ubiquitin carboxyl-terminal hydrolase isozyme L5 |
| NUP88 | Nuclear pore complex protein Nup88 |
| LMNA | Prelamin-A/C |
| SRP68 | Signal recognition particle subunit SRP68 |
| PGK2 | Phosphoglycerate kinase 2 |
| PUS1 | tRNA pseudouridine synthase A |
| PTPN1 | Tyrosine-protein phosphatase non-receptor type 1 |
| YARS2 | Tyrosine--tRNA ligase, mitochondrial |
| RIOX2 | Ribosomal oxygenase 2 |
| DUT | Deoxyuridine 5'-triphosphate nucleotidohydrolase, mitochondrial |
| ARFIP1 | Arfaptin-1 |
| HELLS | Lymphoid-specific helicase |
| CDK2 | Cyclin-dependent kinase 2 |
| CTNNBL1 | Beta-catenin-like protein 1 |
| GPI | Glucose-6-phosphate isomerase |
| DEGS1 | Sphingolipid delta(4)-desaturase DES1 |
| SUCLG2 | Succinate--CoA ligase [GDP-forming] subunit beta, mitochondrial |
| ST13P4 | Putative protein FAM10A4 |
| COPB2 | Coatomer subunit beta' |
| ACAD9 | Complex I assembly factor ACAD9, mitochondrial |
| CCDC47 | PAT complex subunit CCDC47 |
| NCAPH | Condensin complex subunit 2 |
| CNOT1 | CCR4-NOT transcription complex subunit 1 |
| SAMHD1 | Deoxynucleoside triphosphate triphosphohydrolase SAMHD1 |
| CCAR2 | Cell cycle and apoptosis regulator protein 2 |
| POLR2C | DNA-directed RNA polymerase II subunit RPB3 |
| BLVRB | Flavin reductase (NADPH) |
| TFCP2 | Alpha-globin transcription factor CP2 |
| LYAR | Cell growth-regulating nucleolar protein |
| C9 | Complement component C9 |
| RPL31 | 60S ribosomal protein L31 |
| CDK5RAP3 | CDK5 regulatory subunit-associated protein 3 |
| POLA1 | DNA polymerase alpha catalytic subunit |
| CC2D1A | Coiled-coil and C2 domain-containing protein 1A |
| LARP4 | La-related protein 4 |
| STIM1 | Stromal interaction molecule 1 |
| DHX36 | ATP-dependent DNA/RNA helicase DHX36 |
| QRICH1 | Transcriptional regulator QRICH1 |
| AFDN | Afadin |
| PRPSAP2 | Phosphoribosyl pyrophosphate synthase-associated protein 2 |
| RAB10 | Ras-related protein Rab-10 |
| DHRS7B | Dehydrogenase/reductase SDR family member 7B |
| TOMM34 | Mitochondrial import receptor subunit TOM34 |
| SEC13 | Protein SEC13 homolog |
| MRPS9 | 28S ribosomal protein S9, mitochondrial |
| CSTF3 | Cleavage stimulation factor subunit 3 |
| SCRIB | Protein scribble homolog |
| SAP18 | Histone deacetylase complex subunit SAP18 |
| FLII | Protein flightless-1 homolog |
| PPME1 | Protein phosphatase methylesterase 1 |
| DHX40 | Probable ATP-dependent RNA helicase DHX40 |
| SPR | Sepiapterin reductase |
| RFC1 | Replication factor C subunit 1 |
| PDS5A | Sister chromatid cohesion protein PDS5 homolog A |
| DCAF13 | DDB1- and CUL4-associated factor 13 |
| SACM1L | Phosphatidylinositol-3-phosphatase SAC1 |
| TBC1D15 | TBC1 domain family member 15 |
| CDC42 | Cell division control protein 42 homolog |
| IDH2 | Isocitrate dehydrogenase [NADP], mitochondrial |
| NUP54 | Nucleoporin p54 |
| SUCLG1 | Succinate--CoA ligase [ADP/GDP-forming] subunit alpha, mitochondrial |
| PMPCB | Mitochondrial-processing peptidase subunit beta |
| DDX52 | Probable ATP-dependent RNA helicase DDX52 |
| DCAF7 | DDB1- and CUL4-associated factor 7 |
| XRN2 | 5'-3' exoribonuclease 2 |
| ACACA | Acetyl-CoA carboxylase 1 |
