# Supplementary material for: Immunity of turbot Induced by inactivated vaccine of Aeromonas salmonicida from the perspective of DNA methylation
Source: Front Immunol. 2023 Feb 7;14:1124322. doi: 10.3389/fimmu.2023.1124322 (PMC9945314; doi:10.3389/fimmu.2023.1124322)
Supplement: Supplementary file 2 [file Table_2.docx]

| SmC5ar1-Like specific binding proteins | Description |
| --- | --- |
| USP15 | Ubiquitin carboxyl-terminal hydrolase 15 |
| EIF2B2 | Translation initiation factor eIF-2B subunit beta |
| HRNR | Hornerin |
| PLOD1 | Procollagen-lysine,2-oxoglutarate5 dioxygenase 1 |
| FLG | Filaggrin |
| SNRPF | Small nuclear ribonucleoprotein F |
| LGALS7 | Galectin-7 |
| TLN1 | Talin-1 |
| GLUD1 | Glutamate dehydrogenase 1, mitochondrial |
| PAK1 | Serine/threonine-protein kinase PAK 1 |
| SLC7A5 | Large neutral amino acids transporter small subunit 1 |
| UQCRFS1 | Cytochrome b-c1 complex subunit Rieske, mitochondrial |
| PCBP3 | Poly(rC)-binding protein 3 |
| TMX1 | Thioredoxin-related transmembrane protein 1 |
| IGHM | Immunoglobulin heavy constant mu |
| MTIF2 | Translation initiation factor IF-2, mitochondrial |
| ASPH | Aspartyl/asparaginyl beta-hydroxylase |
| GIGYF2 | GRB10-interacting GYF protein 2 |
| MAGED2 | Melanoma-associated antigen D2 |
| KPNA1 | Importin subunit alpha-5 |
| RPAP3 | RNA polymerase II-associated protein 3 |
| BMS1 | Ribosome biogenesis protein BMS1 homolog |
| MRPL22 | 39S ribosomal protein L22, mitochondrial |
| CLTCL1 | Clathrin heavy chain 2 |
| CNOT2 | CCR4-NOT transcription complex subunit 2 |
| MYCL | Protein L-Myc |
| SEPT9 | Septin-9 |

**Supplementary Table S2**. List of 27 the SmC5ar1-Like specific binding proteins.
